# Feasibility of Topical Applications of Natural High-Concentration Capsaicinoid Solutions in Patients with Peripheral Neuropathic Pain: A Retrospective Analysis

**DOI:** 10.1155/2016/9703036

**Published:** 2016-12-27

**Authors:** Fanny Bauchy, Andre Mouraux, Ronald Deumens, Marjolein Leerink, Antonio Ulpiano Trillig, Bernard le Polain de Waroux, Arnaud Steyaert, Quetin-Leclercq Joëlle, Patrice Forget

**Affiliations:** ^1^Department of Anesthesiology, Cliniques Universitaires Saint-Luc, Brussels, Belgium; ^2^Institute of NeuroScience, Cliniques Universitaires Saint-Luc, Brussels, Belgium; ^3^Louvain Drug Research Institute, Cliniques Universitaires Saint-Luc, Brussels, Belgium; ^4^Vrije Universiteit Brussel (VUB), Universitair Ziekenhuis Brussel (UZ Brussel), Brussels, Belgium

## Abstract

*Background.* Capsaicin, one of several capsaicinoid compounds, is a potent TRPV1 agonist. Topical application at high concentration (high concentration, >1%) induces a reversible disappearance of epidermal free nerve endings and is used to treat peripheral neuropathic pain (PNP). While the benefit of low-concentration capsaicin remains controversial, the 8%-capsaicin patch (Qutenza®, 2010, Astellas, Netherlands) has shown its effectiveness. This patch is, however, costly and natural high-concentration capsaicinoid solutions may represent a cheaper alternative to pure capsaicin.* Methods.* In this retrospective study, 149 patients were screened, 132 were included with a diagnosis of neuropathic pain, and eighty-four were retained in the final analyses (median age: 57.5 years [IQR25–75: 44.7–67.1], male/female: 30/54) with PNP who were treated with topical applications of natural high-concentration capsaicinoid solutions (total number of applications: 137). Indications were postsurgical PNP (85.7%) and nonsurgical PNP (14.3%) (posttraumatic, HIV-related, postherpetic, and radicular PNP).* Objectives.* To assess the feasibility of topical applications of natural high-concentration capsaicinoid solutions for the treatment of PNP.* Results.* The median treated area was 250 cm^2^ [IQR25–75: 144–531]. The median amount of capsaicinoids was 55.1 mg [IQR25–75: 28.7–76.5] per plaster and the median concentration was 172.3 *μ*g/cm^2^ [IQR25–75: 127.6–255.2]. Most patients had local adverse effects on the day of treatment, such as mild to moderate burning pain and erythema. 13.6–19.4% of the patients experienced severe pain or erythema. Following treatment, 62.5% of patients reported a lower pain intensity or a smaller pain surface, and 35% reported a sustained pain relief lasting for at least 4 weeks.* Conclusion.* Analgesic topical treatment with natural high-concentration capsaicinoid is feasible and may represent a low cost alternative to alleviate PNP in clinical practice.

## 1. Introduction 

Peripheral neuropathic pain (PNP) results from disease, damage, or dysfunction of the somatosensory system [1]. Its multiple aetiologies include postsurgical neuropathies, posttraumatic neuropathies, painful diabetic polyneuropathies, postherpetic neuralgia (PHN), and HIV-associated neuropathies (HIV-AN) [1]. Epidemiologic data from Europe show that 7–10% of the population is affected by pain with neuropathic characteristics [2]. Nowadays it remains a difficult condition to treat [3] and current systemic treatments like tricyclic antidepressants, serotonin/norepinephrine reuptake inhibitors, anticonvulsants, or opioids provide only moderate pain relief while causing several adverse drug reactions like nausea, dizziness, and somnolence [4]. Topical treatment with capsaicin has been suggested as an interesting alternative way to treat PNP, because it has few significant systemic effects [5], and presents only rare adverse reactions except for local-application burning pain and erythema [6].

The genus* Capsicum* includes species commonly called “pepper” plants and “chilli” peppers. The first recorded use dates back to 3000 BC [7]. The spiciness is due to lipophilic chemicals called capsaicinoids of which the most potent is capsaicin (8-methyl-*N*-vanillyl-6-nonenamide). Other related compounds, found in chilli peppers, include dihydrocapsaicin, homodihydrocapsaicin, homocapsaicin, nordihydrocapsaicin, and nonivamide [8]. Since topical application of capsaicinoids produces a burning sensation, which has been widely used in animal research on models of chemically induced pain,* Capsicum* extracts have also been used as a topical analgesic in traditional medicine. In some countries, low concentration (<1%) of capsaicin creams is available over the counter for muscle or rheumatism, but several daily applications for months are required, thereby creating concerns with efficacy and compliance [9].

The pharmaceutical effects of topical capsaicin applications are based on their action as agonists of Transient Receptor Potential Vanilloid receptor, subtype 1 (TRPV1), which is a ligand-controlled, nonselective cation channel expressed on nociceptive fibers [10]. TRPV1s are heat-sensitive receptors, activated at temperatures between 37 and 45°C. Expressed at the level of free nerve endings, their activation causes a burning pain sensation. TRPV1s can also be activated at basal temperatures when they are bound by exogenous ligands such as capsaicin [11]. Nerve damage causes the upregulation of TRPV1s expression in injured C-fibers, which leads to a pathological amplification in the transduction of heat signals, interpreted as pain [11]. A single application of high-concentration capsaicin or repeated applications of low concentration triggers a desensitization of TRPV1s-expressing free nerve endings through a yet unexplained mechanism. This leads to a temporary retraction or destruction of epidermal free nerve endings. The result is a reduction of nociceptive input from the treated region, possibly leading to a reduction of pain in PNP [12, 13]. However, topical application of capsaicin also causes a transient local erythema due to the release of neuropeptides during the capsaicin-induced neurogenic inflammation [14].

High-concentration patches of pure capsaicin 8% (Qutenza, Astellas) have been available in most European countries since 2009. The European Union approved the patches for the treatment of PNP in nondiabetic adults, to be used either alone or in combination with other medical products [15]. It has been studied in several clinical trials for PHN [16, 17], HIV-AN [18, 19], in prospective analysis for general PNP [6]. These trials have all shown significant 30–40% reductions in pain scores that were maintained over an 8-week period after a single application, although the high cost of the patches (over 300€ per patch in Belgium) currently limits their clinical use.

This retrospective analysis with commercially available natural high-concentration capsaicinoid solutions aims to determine the feasibility of using these as an alternative low cost topical treatment for PNP.

## 2. Materials and Methods

This study was conducted in the Pain Clinic of the Saint-Luc University Hospital (Cliniques Universitaires Saint-Luc, Brussels, Belgium) with the agreement of the ethical committee (Ethical number approval: 2014/20MAI/267, chairperson: Prof. Jean-Marie Maloteaux). It took place between January 2013 and December 2014 and involved 84 patients. All patients were treated with an application of natural high-concentration capsaicinoid solution. Only patients suffering from definite PNP (according to the IASP criteria, first described by Treede et al.) were considered. These criteria are plausible neuroanatomical distribution, appropriate history, altered sensory signs, and evidence of lesion or disease [20]. We included patients being 17 years old and older and patients with diabetes. Patients were excluded (*n* = 48) if they were found not to suffer from PNP, a Qutenza patch was previously used, the medical file was incomplete, and the follow-up was missing. As this study is a retrospective analysis, all the patients were treated according to their need, independently of this study.

Two different natural high-concentration capsaicinoid solutions were used (Chilli extract, Wirrmann, UK). As their exact contents were not given by the manufacturers, we decided to have them analysed using High-Performance Liquid Chromatography (HPLC) coupled to a UV detector at the Pharmacognosy Research Group of the University (Louvain Drug Research Institute, LDRI, Brussels, Belgium). The HPLC was performed following a validated methodology described in the European pharmacopeia (capsici oleoresina raffinata et normata) [21] using a LaChrom Elite HPLC integrated system (Merck Hitachi, VWR, Leuven, Belgium) equipped with a L-2450 UV detector, a L-2300 oven, L-2130 autosampler, and L-2130 pump, with EZChrom software. Isocratic elution was used with Agilent Zorbax® Eclipse XDB phenyl 250 mm × 4.6 mm column (5 *μ*M). The HPLC analysis of the capsicum extracts showed that the first solution used contained 0.71% of total capsaicinoid and the second solution 2.01%.

Patients were treated with a plaster gauze, adjusted to the painful area previously drawn on the skin. Before applying the gauze onto the skin, it was impregnated with capsaicinoid solution. Ten patients were treated with the first solution and 53 with the second one. The median area per application was 250 cm^2^ [IQR25–75: 144–531] and the median amounts of solution per application were 100 drops [IQR25–75: 50–155], or 2.8 mL [IQR25–75: 1.4–4.4]. The skin region to be treated was always intact and dried before the plaster was applied by an anaesthesiologist. An intravenous catheter was placed before treatment for safety and pain management. Patients were premedicated with 5% lidocaine gel on the target skin, lidocaine-infiltration, or tramadol 100 mg, performed 60 minutes before plaster application. After application, the plaster remained on the target skin for 60 minutes. Elastic bandages were used to improve adhesion between the skin and plaster. After removal, the skin was cleaned with a cleaning gel and cooled with a cold pack. The duration of cooling depended on the patient's needs and patients were recommended to continue its use at home for several days, if necessary. Where the cold pack proved to be insufficient, treatment-related discomfort was alleviated before going home with paracetamol 1 g, tramadol 50 mg, or titrated IV morphine. Any adverse events were reported and followed up until they were resolved and explained. After the initial visit, follow-up visits were offered to the patients for several weeks. A second capsaicinoid plaster was applied at the next visit to the hospital if the pain threshold had risen again after the initial relief, even if it was not as intense as the first. A majority of the study subjects had concomitant chronic medications. Some patients had painkillers which were classified based on the analgesic ladder which has three steps: paracetamol and nonsteroidal anti-inflammatory drugs belong to the first step, tramadol and tilidine-naloxone belong to the second, and morphine belongs to the third. Other patients had current systemic treatments like tricyclic antidepressants, serotonin/norepinephrine reuptake inhibitors, or anticonvulsants. Those chronic medications were kept and reevaluated throughout the follow-up.

Pain levels were assessed before the treatment using a 0–10 Visual Analog Scale (VAS). Presence of neuropathic symptoms was assessed clinically by an anaesthesiologist (French version of the DN4 questionnaire) [22]. Pain was evaluated immediately after treatment to decide if a treatment-related discomfort therapy was necessary. The pain characteristics to the application were estimated during the follow-up visits. We asked patients whether they had experienced a significant pain reduction in terms of intensity and/or surface area over time and asked them about their general impression of pain change in terms of being positive (verbalized as good) or negative (bad).

To assess the potential of the treatment, data from subgroups of patients were analysed for differences in terms of global impression of pain change, lower pain intensity, and surface area after the first high-concentration capsaicinoid plaster application. The cohorts of patients compared were based on (i) the DN4 (DN4 < 4 versus DN4 ≥ 4); (ii) the pain origins (non-post-surgical PNP versus postsurgical PNP; post-total knee replacement PNP versus other PNP; post-knee-surgery PNP versus other PNP); (iii) the premedication given (tramadol versus other premedications); (iv) the pain intensity and erythema reaction after application (none or mild pain versus medium or strong pain); and (v) the concentration of capsaicinoids given (<200 *μ*g/cm^2^ or ≥200 *μ*g/cm^2^).

Biometrical analysis followed statistical analysis rules. Data were described as mean ± SD and median [IQR25–75] when appropriate. Proportion, in percentage, was expressed with 95% confidence interval (95% CI). Subgroup differences, in terms of pre- and posttreatment pain scores per patient, were analysed using Student's qualitative* t-*test (paired *t*-test) for independent-sample comparisons. Statistical analysis was performed using R language (version 3.2.0). The threshold for statistical significance level was set at *p* < 0.05.

## 3. Results

One hundred and forty-nine patients were considered for inclusion in this study. Seventeen were excluded for not meeting the inclusion criteria regarding the diagnosis of neuropathic pain. One hundred and thirty-two patients received a total number of 291 applications of the natural high-concentration capsaicinoid. Of these patients, evaluation files were completed for 137 applications from 84 patients, 30 of whom were males (35.7%) and 54 were females (64.3%). The median age was 57.5 years [IQR25–75: 44.7–67.1]. The median baseline VAS pain score was 6 [IQR25–75: 5–7]. In seventy-three patients, the DN4 questionnaire was complete, with a median baseline DN4 score of 4 [IQR25–75: 3–6]. Fifty-one patients scored ≥4, and twenty-four scored <4 (in which 4 patients scored 2). Of these patients, 14.3% had nonsurgical PNP while 85.7% had postsurgical PNP. 52.4% of the previous group received a knee surgery. The population is further described in Table 1.

**Figure 1:**
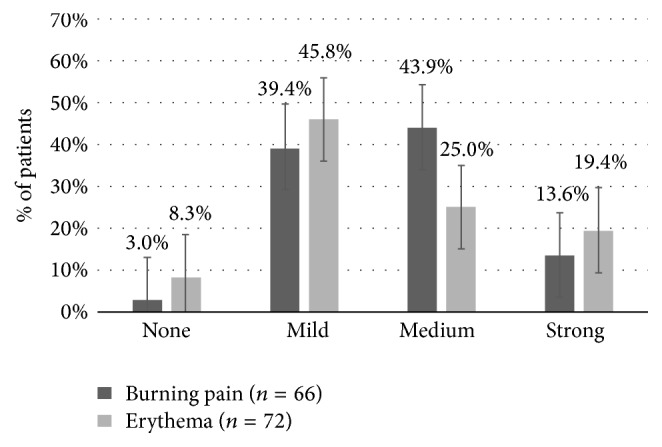
High-concentration capsaicinoid application tolerability immediately after treatment. Proportion (%) of treatment-related discomfort in terms of burning pain (66 patients) or erythema in (72 patients) ranked in order of intensity (IC = 95%).

Based on the DN4 score, patients were asked to describe their PNP and the results recorded on files. The most-often used descriptors for pain were burning (73.2%), tingling (73.2%), and pins and needles (62.5%). Clinical examination indicated a tactile hyperesthesia, hyperalgesia, or allodynia in 91.0% of patients (Table 2). Several concomitant medications were taken by most of the patients for chronic PNP before high-concentration capsaicinoid application with 50.6% of patients having first-step painkillers, 40.0% having second-step painkillers, 11.8% having third-step painkillers, 25.9% having anticonvulsants, and 15.3% having antidepressants. Only 5.9% of patients had no medication for chronic pain (Table 3). Nonpharmacological pain therapies were not reported. Taking into consideration the quantity of capsaicinoid solution applied over the plasters, the median capsaicinoid concentration was 172.3 *μ*g/cm^2^ [IQR25–75: 127.6–255.2], with a median of 55.1 mg [IQR25–75: 28.7–76.5] of capsaicinoids per plaster. Before the high-concentration capsaicinoid application, 85.4% of patients received tramadol 100 mg (*n* = 41) while 8.3% had another premedication such as topical-lidocaine (*n* = 1) and local anaesthetic infiltration (*n* = 2). 6,3% (*n* = 3) did not receive any premedication. Immediately after the application, evaluation showed that most of the 132 patients had local adverse effects with 83.3% of patients reporting mild or medium burning pain (*n* = 66), 70.8% having mild or medium erythema (*n* = 72), and only 13.6% having strong burning pain and 19.4% having strong erythema (Figure 1). Treatment-related side effects required a cold pack in 69.8% of the cases. Only 7.0% of patients required an additional second- or third-step painkiller.

**Figure 2:**
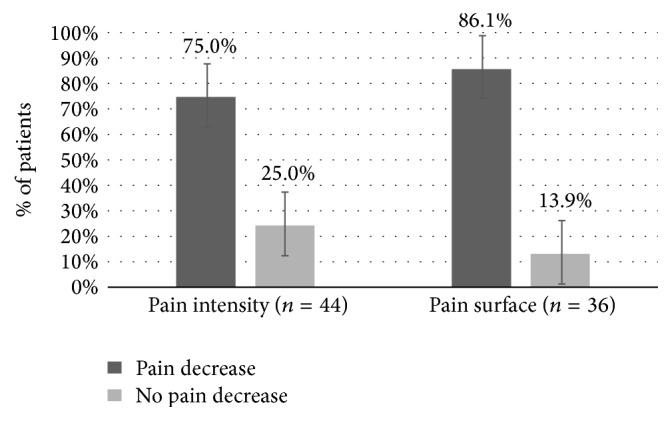
Peripheral neuropathic pain evolution, weeks after high-concentration capsaicinoid treatment. Proportion (%) of change in terms of pain intensity (44 patients) and pain surface area (36 patients) in the group of satisfied patients (IC = 95%).

The follow-up visits, several weeks after the application, showed that 62.5% of the 132 patients had a positive global impression of pain change (*n* = 80). Within this group, 75.0% reported a decrease in their pain intensity (*n* = 44) and 86.1% reported a decrease in their pain surface area (*n* = 36) (Figure 2). We did not identify any predictive factor for the effect of the treatment after looking for differences between the subgroups previously described, especially regarding the value of the DN4, or the presence of a score more or less than 4 before the treatment (*p* > 0.05 for all the comparisons).

## 4. Discussion

This analysis confirms the feasibility of natural high-concentration capsaicinoid applications as a treatment of PNP in clinical practice. As a retrospective study, this analysis is limited by the lack of a control group and can therefore not be used for the assessment of treatment effectiveness.

The population sample was relatively homogeneous with 85.7% of patients suffering from postsurgical PNP. This homogeneity is due to the fact that most of our patients were being sent by orthopaedic and cardiovascular surgeons. However, even if all the patients met the IASP criteria for definite neuropathic pain, a recent consensus of the NeuPSIG group report that, without additional investigations, the presence of neuropathic pain can only be seen as “probable” to “definite” [23]. Of note, seeing the presence of an “appropriate history” and the “presence of lesion or disease,” we considered a high a priori probability for the presence of a neuropathic pain. Thus, we used the DN4 questionnaire as an additional assessment tool and found a DN4 less than 4 in a significant proportion of the patients, which is questionable. But in most of these patients, only one item was missing, and some of the patients with a missing DN4 may have higher scores. Anyway, we did not find any difference in terms of efficacy of the treatment associated with DN4 criteria, as being DN4 < 4 versus DN4 ≥ 4.

Despite some clear variability in our results, the quantity of capsaicinoids per application was relatively high because of the large amounts of solution used to impregnate the plasters (about 2.8 mL per application). We believe this variability may be easily reduced by establishing a standardised table of number of drops per standard-sized plaster (drop/cm^2^). In comparison to our median amounts of capsaicinoids per application of 55.1 mg [IQR25–75: 28.7–76.5], the high-concentration Qutenza has a concentration of 640 *μ*g/cm^2^ and 179 mg of capsaicin per plaster [24], and the low-concentration Zostrix® cream 0.075% has around 3 mg of capsaicin per application [25], which makes our application 3.2 less strong than the high-concentration one but at least 18.2 times stronger than the low-concentration one (Table 4).

The application of high-concentration capsaicinoids was generally well-tolerated, with 6.9% of patients needing a treatment-related-discomfort medication to alleviate the side effects in comparison to the 15–44% often reported in clinical trials [6, 16–19]. Only burning sensations and erythema were reported as side effects, the treatment of which was easily manageable.

Although the aim of our study was not to assess the efficacy of high-concentration capsaicinoids and the results did not have enough power to allow a statistical assessment of clinical efficacy, the emerging trends for pain reduction in global impression of change observed in this study seem to be comparable to that found in previous RCTs on high-concentration capsaicin [6, 16–19]. One important difference with the commercially available high-concentration capsaicin patch (Qutenza, Astellas) is the cost. Indeed, even if available from 2009 in Europe, the high cost (more than 300€) without reimbursement in some countries (as in Belgium) renders its use nonpracticable. We did not performed cost-effectiveness analysis. But the demonstrated feasibility of the use of present alternative (natural extract, costing only a few euros), warrants further investigations, especially to facilitate the quality control before potential diffusion.

## 5. Conclusions

In conclusion, this retrospective study showed that topical treatment of PNP using natural high-concentration capsaicinoid applications is feasible, with generally mild to moderate and short-lasting side effects. This low cost alternative may constitute a new opportunity to widen the topical treatments available for patients suffering from PNP.

## Figures and Tables

**Table 1:** Characteristics of patients (*n* = 84).

Demographical and clinical data	Value	
Patients	84	
Male, *n* (%)	30 (35.7%)	
Female, *n* (%)	54 (64.3%)	
Age, years, median [IQR25–75]	57.5 [44.5–67.1]	
Baseline VAS score, median [IQR25–75]	6 [5–7]	
Baseline DN4 score, median [IQR25–75]	4 [3–6]	

Polyneuropathies diagnosis	*n*	%

(I) Non-post-surgical PNP		
(a) Posttraumatic neuropathy without surgery	4	4.8
(b) HIV-associated neuropathy	1	1.2
(c) Foot diabetic polyneuropathy	1	1.2
(d) Radiculopathy	2	2.4
(e) Postherpetic neuralgia	2	2.4
(f) Other	2	2.4
Total of nonsurgical PNP	**12**	**14.3**
(II) Postsurgical PNP		
(a) Postthoracotomy or thoracoscopy	9	10.7
(b) Postparietal surgery	3	3.6
(c) Postabdominal surgery	4	4.8
(d) Postback surgery	5	6.0
(e) Other	3	3.6
(f) Posttraumatic surgery (except knee)	4	4.8
(g) Posttotal knee replacement	31	36.9
(h) Posttraumatic knee surgery	4	4.8
(i) Post-other knee surgery	9	10.7
Total of knee surgeries	44	52.4
Total of postsurgical neuropathies	**72**	**85.7**

Localizations of pain, multiple responses possible	*n*	%

Knee	45	53.6
Hemithorax	13	15.5
Foot	9	10.7
Back	5	6.0
Abdomen	4	4.8
Other	4	4.8
Leg	3	3.6
Ankle	3	3.6

**Table 2:** Patients' pain descriptions and pain clinical examinations at baseline.

Patients' descriptions at baseline	*n*	%
(i) Burning (*n* = 52)	41	73.2
(ii) Tingling (*n* = 50)	41	73.2
(iii) Pins and needles (*n* = 44)	35	62.5
(iv) Electric shock sensation (*n* = 47)	33	58.9
(v) Numbness (*n* = 41)	29	51.8
(vi) Itching (*n* = 33)	17	48.5
(vii) Waking-up pain (*n* = 46)	25	44.6
(viii) Painful cold sensation (*n* = 34)	21	37.5

Clinical examinations at baseline		

Hypoesthesia/hypoalgesia:		
(i) Tactile (*n* = 42)	28	66.7
(ii) Pricking (*n* = 38)	24	63.2
(iii) Thermal (*n* = 43)	25	58.1
Hyperesthesia/hyperalgesia/allodynia		
(i) Tactile (*n* = 67)	61	91.0
(ii) Thermal (*n* = 25)	24	61.5
(iii) Pricking (*n* = 24)	21	56.8

**Table 3:** Concomitant medications for PNP at baseline (*N* = 84), multiple responses possible.

	*n*	%
First-step painkillers	43	50.6
Second-step painkillers	34	40.0
Third-step painkillers	10	11.8
Anticonvulsants	22	25.9
Antidepressants	13	15.3
Other medications	10	11.8
No medication	5	5.9

**Table 4:** Characteristics of high-concentration capsaicinoid applications.

Median capsaicinoid concentration per application	*μ*g/cm^2^	[IQR25–75]
(i) In 2013 (*n* = 10)	90.0	[51.0–123.0]
(ii) In 2014 (*n* = 53)	255.0	[144.0–262.5]
(iii) Total (*n* = 63)	172.3	[126.7–255.2]

Median weight of capsaicinoid per application	mg	

(i) In 2013 (*n* = 10)	51.0	[25.0–71.0]
(ii) In 2014 (*n* = 53)	55.0	[29.0–75.5]
(iii) Total (*n* = 63)	55.1	[28.7–76.5]

Median surface area per application, cm^2^ (*n* = 67)	250.0	[144.0–531.0]
Median amount of mL per application, mL (*n* = 63)	100.0	[1.4–4.4]

## References

[1] Baron R., Binder A., Wasner G. (2010). Neuropathic pain: diagnosis, pathophysiological mechanisms, and treatment. *The Lancet Neurology*.

[2] Van Hecke O., Austin S. K., Khan R. A., Smith B. H., Torrance N. (2014). Neuropathic pain in the general population: a systematic review of epidemiological studies. *Pain*.

[3] Attal N. (2012). Neuropathic pain: mechanisms, therapeutic approach, and interpretation of clinical trials. *CONTINUUM Lifelong Learning in Neurology*.

[4] O'Connor A. B., Dworkin R. H. (2009). Treatment of neuropathic pain: an overview of recent guidelines. *American Journal of Medicine*.

[5] Horváth K., Boros M., Bagoly T. (2014). Analgesic topical capsaicinoid therapy increases somatostatin-like immunoreactivity in the human plasma. *Neuropeptides*.

[6] Maihofner C., Heskamp M.-L. (2013). Prospective, non-interventional study on the tolerability and analgesic effectiveness over 12 weeks after a single application of capsaicin 8% cutaneous patch in 1044 patients with peripheral neuropathic pain: first results of the QUEPP study. *Current Medical Research and Opinion*.

[7] Schumacher M. (2010). TRP channels in pain and inflammation: therapeutic opportunities. *Pain Practice*.

[8] Luo X.-J., Peng J., Li Y.-J. (2011). Recent advances in the study on capsaicinoids and capsinoids. *European Journal of Pharmacology*.

[9] Peppin J. F., Pappagallo M. (2014). Capsaicinoids in the treatment of neuropathic pain: a review. *Therapeutic Advances in Neurological Disorders*.

[10] Cortright D. N., Szallasi A. (2009). TRP channels and pain. *Current Pharmaceutical Design*.

[11] Spicarova D., Palecek J. (2008). The role of spinal cord vanilloid (TRPV1) receptors in pain modulation. *Physiological Research*.

[12] Knotkova H., Pappagallo M., Szallasi A. (2008). Capsaicin (TRPV1 agonist) therapy for pain relief: farewell or revival?. *Clinical Journal of Pain*.

[13] Malmberg A. B., Mizisin A. P., Calcutt N. A., Von Stein T., Robbins W. R., Bley K. R. (2004). Reduced heat sensitivity and epidermal nerve fiber immunostaining following single applications of a high-concentration capsaicin patch. *Pain*.

[14] Winter J., Bevan S., Campbell E. A. (1995). Capsaicin and pain mechanisms. *British Journal of Anaesthesia*.

[15] McCormack P. L. (2010). Capsaicin dermal patch: in non-diabetic peripheral neuropathic pain. *Drugs*.

[16] Irving G. A., Backonja M. M., Dunteman E. (2011). A multicenter, randomized, double-blind, controlled study of NGX-4010, a high-concentration capsaicin patch, for the treatment of postherpetic neuralgia. *Pain Medicine*.

[17] Backonja M., Wallace M. S., Blonsky E. R. (2008). NGX-4010, a high-concentration capsaicin patch, for the treatment of postherpetic neuralgia: a randomised, double-blind study. *The Lancet Neurology*.

[18] Simpson D. M., Gazda S., Brown S. (2010). Long-term safety of NGX-4010, a high-concentration capsaicin patch, in patients with peripheral neuropathic pain. *Journal of Pain and Symptom Management*.

[19] Clifford D. B., Simpson D. M., Brown S. (2012). A randomized, double-blind, controlled study of NGX-4010, a capsaicin 8% dermal patch, for the treatment of painful hiv-associated distal sensory polyneuropathy. *Journal of Acquired Immune Deficiency Syndromes*.

[20] Treede R.-D., Jensen T. S., Campbell J. N. (2008). Neuropathic pain: redefinition and a grading system for clinical and research purposes. *Neurology*.

[21] EDQM (2014). *The European Pharmacopea*.

[22] Bouhassira D., Attal N., Alchaar H. (2005). Comparison of pain syndromes associated with nervous or somatic lesions and development of a new neuropathic pain diagnostic questionnaire (DN4). *Pain*.

[23] van Hecke O., Kamerman P. R., Attal N. (2015). Neuropathic pain phenotyping by international consensus (NeuroPPIC) for genetic studies: a NeuPSIG systematic review, Delphi survey, and expert panel recommendations. *Pain*.

[24] Astellas Pharma (2014). *Qutenza 179 Mg Cutaneous Patch*.

[25] AFT Pharmaceuticals (2011). *Zostrix HP, Capsaicin USP 0.075% w/w Topical Cream*.

